# Social stigma and familial attitudes related to infertility

**DOI:** 10.4274/tjod.04307

**Published:** 2018-03-29

**Authors:** Rahime Nida Ergin, Aslıhan Polat, Bülent Kars, Deniz Öztekin, Kenan Sofuoğlu, Eray Çalışkan

**Affiliations:** 1 Bahçeşehir University Faculty of Medicine, Department of Obstetrics and Gynecology, İstanbul, Turkey; 2 Kocaeli University Faculty of Medicine, Department of Psychiatry, İzmit, Turkey; 3 Kartal Dr. Lütfi Kırdar Training and Resarch Hospital, Clinics of Obstetrics and Gynecology, İstanbul, Turkey; 4 İzmir Aegean Materniti and Women’s Diseases Training and Resarch Hospital, Clinics of Obstetrics and Gynecology, İzmir, Turkey; 5 Zeynep Kamil Women and Children’s Dieases Training and Resarch Hospital, Clinics of Obstetrics and Gynecology, İstanbul, Turkey

**Keywords:** Infertility, intra-cytoplasmic sperm injection, familial attitudes, social stigma

## Abstract

**Objective:**

To determine the perceived social stigma and familial attitides and perception of sexuality in infertile couples attending infertility clinics.

**Materials and Methods:**

Infertile couples attending infertility clinics between the years of 2014 and 2015 were requested to complete detailed evaluation forms including questions related to the social stigma on their infertility, their familial attitudes, and perception of sexuality. Any partner of the infertile couple accepting to enroll in the study was given the evaluation forms. Their scores related to answers and demographics, and parameters related to infertility were analyzed.

**Results:**

A total 598 partners of infertile couples enrolled in the study, 58% represented 177 couples. Their infertility was primary in 98.3% and the duration of marriage and infertility was 9.81±5.58 and 9.76±5.53 years, respectively. The perception of social exclusion was present in 38% (p<0.001) of infertile couple, which was more significantly pronounced in female partners (p=0.013). Fifteen percent of the infertile couples thought themselves as isolated in public and losing value in public (p<0.001). However, sixty percent of infertile couples thought that they would achieve a notable place in community after having a baby (p<0.001). Infertility was accepted as a reason of divorce in only 13% of infertile couples (p<0.001). The majority of perticipnats, irrespective of sex, rejected that infertile women or men lost sexual appeal (86%; p<0.001).

**Conclusions:**

There is significant effect of infertility on familial attitudes and perception of sexuality of infertile couples. Unfortunately, there is significant negative social stigma on infertile couples.

**PRECIS:** Infertility itself has negative effect on couples in terms of social stigma and familial attitudes, as well as sexuality.

## Introduction

Infertility is a worldwide health issue with approximately 49 million couples affected in 2010^(1)^. Within the last two decades, there has not been a significant change in the rates of infertility worldwide, regardless of populational growth, despite the development of technical and medical improvements in diagnostic and surgical techniques as well as summated experience on this subject^(1)^. In all communities worldwide, infertility is an important issue for both sexes because it is an instinctive biologic behavior to have an offspring, but also an important issue to be a family as a part of a community. However, infertility has recently gained attention to its psychological aspects such as depression and sexual dysfunction, and the negative effects on partner relationship with varying severities and rates^(2,3,4,5,6,7,8)^. Therefore, it is also an important issue for medical caregivers working in infertility, to deal with and determine the psychological aspects of infertility. In this prospective questionnaire and interview-based clinical study, we aimed to determine the effect of infertility on familial attitudes, the perception of sexuality of infertile couples, as well as the effect of the infertility-driven social stigma on infertile couples attending infertility clinics. In addition, we aimed to underline the necessity for psychological support for the psychological aspects of infertility beyond its medical management.

## Materials and Methods

A prospective study using questionnaire forms conducted in a referral centers for assisted reproduction was conducted after approval of the local ethics committee. After obtaining written informed consent, infertile couples attending infertility clinics between the years of 2014 and 2015 were requested to complete detailed evaluation forms including questions related to the social stigma on their infertility, their familial attitudes, and perception of sexuality together with the Golombok-Rust Inventory of Sexual Satisfaction, Rosenberg’s Self-Esteem Scale, and Beck’s Depression Inventory^(9,10,11)^. Any partner of the infertile couple accepting to enroll in the study was given the questionnaire forms. Their scores related to answers and demographics, and parameters related to infertility were analyzed.

### Statistical Analysis

The related statistical comparisons of groups were performed with the ANOVA test and chi-square test where appropriate. Correlation analyses were performed using Pearson’s correlation. Statistical analyses were performed using SPSS statistics software (SPSS Statistics for Windows, Version 17.0; SPSS Inc., Chicago, U.S.A). p was set as <0.05 for significance.

## Results

A total 598 partners (380 females and 218 male partners) of infertile couples enrolled in the study, 344 (58%) of which represented 177 couples. Their infertility was primary in 98.3% and the duration of marriage and infertility was 9.81±5.58 and 9.76±5.53 years, respectively. In 52%, ≥1 sessions of antiretroviral therapy were applied and pregnancy was achieved in 6% of them previously. Educational status was high school level or higher in 25%. The mean age of the male partners was 35.46±6.26 years and the mean age of the female partners was 32.07±5.44 years. The mean body mass indexes were 26.25±3.23 kg/m^2^ and 25.42±3.35 kg/m^2^ for the male and female partners, respectively. The patients mostly (94%) attended the infertility clinics with their spouses. When informed about the presence of infertility, 46% tended to inform their spouses initially. When compared for sex, male participants tended to inform at first their spouses significantly more compared with the female participants (53% vs 43%, p=0.007) and the ratio of female participants who at first informed their mothers was higher compared with male participants (34% vs 27%, p=0.007). Almost half of the infertile participants (44%) tended to hide this from the community in which they lived. Non-medical applications were applied in 86%, most of which were referral to a herbalist for officinal plants (30%) or so-called “healing water” (12%). 

The perception of social exclusion was present in 38% (p<0.001) of infertile participants, more significantly in female partners (43% in females; 29% in males) (p=0.013). Only fifteen percent of the infertile participants, irrespective of sex, thought that the infertile woman was a second-class person (p<0.001), which was more significantly pronounced among females (19% in females; 10% in males) (p=0.003). On the other hand, in cases of male infertility, this rate dropped to 10% without any significant sex difference. Fifteen percent of the infertile participants thought themselves as isolated in public and losing value (p<0.001). However, 60% of the infertile participants though that they would achieve a notable place in community after having a baby (p<0.001). 

The community was informed about someone’s infertility by infertile individuals or their spouses in 58%, and members of the community tended to spread this information as frequently as 42% (p>0.05), reflecting the importance that the community gives to the issue of infertility. The community members’ attitudes were mostly negative to infertile females compared to males (57% vs 37%; p=0.001). Men tended to give higher scores for their wives’ wish for children than for theirs’ compared with vice versa (p=0.025) (Table 1). Correlations of parameters related to social stigma showed some significant correlations among each other (Table 2).

Most of the infertile participants (72%) mentioned that they would feel similar if their spouses rather than themselves were diagnosed as infertile (p<0.001). Most of them neither supported nor disagreed with adopting a child. Approximately one third (35%) thought that having a child was a must for being a family (p<0.001). Almost half of the infertile particioants did not think that reproduction was the most important mission and more than two-thirds (68%) disagreed that the most important duty of a woman was to give birth (p<0.001). 

Infertility was accepted as a reason of divorce in only 13% of infertile participants (p<0.001), which was more significantly pronounced in female partners (17% in females; 7% in males) (p=0.002). The infertile participants mostly did not wish to have not to been married (89%: p<0.001). However, this rate decreased, albeit still high, to 74% (p<0.001) concerning their thoughts about their spouses’ choice, which was statistically more evident in females (67%) compared with male ones (78%) (p=0.014). The majority of the infertile participants, either men or women, rejected the perception of infertility as being a sign of male impotence (86%; p<0.001). Likewise, the majority, irrespective of sex, rejected that infertile women or men lost sexual appeal (86%; p<0.001).

## Discussion

Infertility is an important worldwide health issue with no significant change in its prevalence within the last two decades^(1)^. Childbearing is an important issue on a personal basis and for the community, which in turn causes unfavorable psychological aspects such as depression and sexual dysfunction, and negative effects on partner relationships with varying severities and rates^(2,3,4,5,6,7,8)^. Surprisingly, depression itself is also a predictor of treatment outcome in artificial reproduction techniques^(12)^. Likewise, stress-related alterations in the hypothalamic-pituitary-gonadal axis has been suggested to result in changes in sexual behavior and in changes in gonadotrophins levels, which in turn may explain the inter-relationship of sexuality, infertility, and stress^(13)^. Therefore, it is important deal with psychological aspects of infertility to break this possible vicious cycle in infertility treatment. To be success in this aim, the first step is to outline all aspects of personal psychology and partner relationships as well as the social stigma. A recent systematic review suggested incongruent results due to different objectives and methodologies, the lack of specific questionnaires to assess sexual function, and uncontrolled social and relationship variables interfering sexual functions, which made it difficult to establish the impact of infertility on the sexual function of infertile couples^(14)^. Another systematic review also suggested that infertility had a negative effect on the psychological well-being and sexual relationships of couples, but the evidence was inconclusive for the effect on familial attitudes and quality of life^(15)^. Therefore, further multicentered studies are warranted to clarify these points. In our study, the behaviours of couples tended to differ even starting from the diagnosis of infertility. That is, infertile men tended to share this initially with their spouses more frequently compared with infertile women. That seems to be related to the higher rates of fear for social exclusion in infertile women and decreased self-esteem resulting from infertility. Regardless of sex, almost half of the infertile couples tended to hide their status of infertility from the commity with the fear of social stigma. In addition, it is confirmed that infertility is an important issue in the community because the community itself spread and evaluated the knowledge of an individual’s infertility as a negative property of that person or couple. Ironically, social support has been suggested to be important for self-esteem and quality of life in infertile couples^(16)^. In this study, it is seen that scores reported for wishing to have a baby were very high for partners of infertile couples and their parents. In the correlation analyses, we found that the desire to have a child showed a weak but positive correlation with advancing infertility period. Correlations of scores reported for wishing to have a baby of infertile couples with those of their parents showed a very strong positive correlation, further indicating the importance of a baby and the possible negative pressure on infertile couples. Interestingly, scores of perceived partners’ wishes of having a baby were reported as low by female partners compared with male partners, which further indicates the presence of more social pressure on infertile female partners. In a similar manner, infertile women tended to accept infertility as a reason for getting divorced more compared with infertile men (17% vs 7%), and almost one-third of infertile women wished not to have been married due to low self-esteem or blame related to infertility, whereas this rate was significantly lower in men (22%). In the present study, we found a negative perception of sexual dysfunction or negative effects on perception of sexual appeal irrespective of sex in infertile couples, though at a lower rate. Nevertheless, these results further confirm that psychological counseling is a need, which can provide valuable assistance in accordance with infertility managements of infertile couples^(17)^.

### Study Limitations

This study needs to be confirmed by further studies with larger numbers of patients.

## Conclusion

There is a significant effect of infertility on familial attitudes and perception of sexuality of infertile couples. 

## Figures and Tables

**Table 1:**
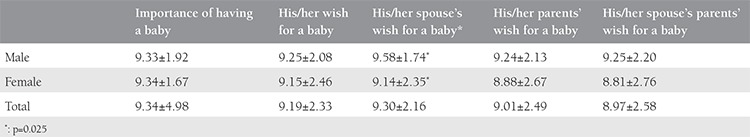
Attitudes of infertile couples and their parents regarding having a baby (scores 0 to 10; 10 indicating the highest rank)

**Table 2:**
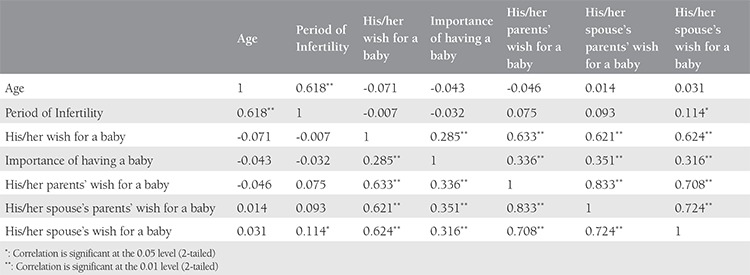
Correlations of age, period of infertility, and attitudes of infertile couples and their parents regarding having a baby
